# A Rare Coexistence of Thyrotropin-Secreting Pituitary Adenoma and Graves Disease

**DOI:** 10.1210/jcemcr/luaf173

**Published:** 2025-07-31

**Authors:** Yuichi Oda, Kosaku Amano, Yasufumi Seki, Daisuke Watanabe, Atsuhiro Ichihara, Takakazu Kawamata

**Affiliations:** Department of Neurosurgery, Tokyo Women's Medical University, Shinjuku-ku, Tokyo 162-8666, Japan; Department of Neurosurgery, Tokyo Women's Medical University, Shinjuku-ku, Tokyo 162-8666, Japan; Department of Medicine, Division of Hormonal Medicine and Bioregulatory Science, Tokyo Women's Medical University, Shinjuku-ku, Tokyo 162-8666, Japan; Department of Medicine, Division of Hormonal Medicine and Bioregulatory Science, Tokyo Women's Medical University, Shinjuku-ku, Tokyo 162-8666, Japan; Department of Medicine, Division of Hormonal Medicine and Bioregulatory Science, Tokyo Women's Medical University, Shinjuku-ku, Tokyo 162-8666, Japan; Department of Neurosurgery, Tokyo Women's Medical University, Shinjuku-ku, Tokyo 162-8666, Japan

**Keywords:** hyperthyroidism, Graves disease, thyrotroph pituitary adenoma, diagnosis

## Abstract

The coexistence of Graves disease (GD) and a thyroid-stimulating hormone (TSH)–secreting pituitary adenoma (TSHoma) is a rare and diagnostically challenging condition. In general, GD typically manifests with low TSH because of excess thyroid hormone production; contrastingly, a TSHoma causes secondary hyperthyroidism with normal or elevated TSH levels. This unusual overlap poses diagnostic and therapeutic challenges; therefore, a careful approach is required to distinguish and manage both conditions. We present the case of a 51-year-old woman with symptoms of hyperthyroidism, elevated thyroid hormones, low TSH, and positive anti-TSH receptor antibodies, which were suggestive of GD 10 years prior. After stopping thiamazole and levothyroxine because of the favorable control of thyroid function 6 months prior, the patient presented a syndrome of inappropriate secretion of TSH and magnetic resonance imaging revealed a pituitary macroadenoma; accordingly, she was diagnosed with concurrent GD and a TSHoma. Treatment involved transsphenoidal resection of the TSHoma and antithyroid medication to control GD. This case illustrates the rarity of coexisting GD and TSHoma and the diagnostic and therapeutic complexities of managing dual hyperthyroidism etiologies. Biochemical testing, antibody assessment, and imaging examination are essential for accurate and early diagnosis of the condition.

## Introduction

Hyperthyroidism is mainly caused by Graves disease (GD) and rarely by pituitary tumors. The coexistence of GD with a thyroid-stimulating hormone (TSH)–secreting pituitary adenoma (TSHoma) is extremely rare. We report a case of GD complicated by a syndrome of inappropriate TSH secretion during a clinical course spanning 10 years, subsequently diagnosed with a coexisting TSHoma.

## Case Presentation

A 51-year-old Japanese woman presented with hand tremor without ophthalmic symptoms. She had been diagnosed with GD at a prior facility in 2014. Biochemical evaluation showed primary hyperthyroidism: TSH, 0.0045 μIU/mL (SI: 0.0045 μIU/mL) (reference, 0.20-4.50 μIU/mL [SI: 0.20-4.50 μIU/mL]); free triiodothyronine (FT3) 9.69 pg/mL (SI: 14.88 pmol/mL) (reference, 2.2-4.3 pg/mL [SI: 3.38-6.60 pmol/mL]); free thyroxine (FT4), 2.26 ng/dL (SI: 29.15 pmol/mL) (reference, 0.80-1.60 ng/dL [SI: 10.32-20.64 pmol/mL]); and anti-TSH receptor antibody (TRAb), 43.6 IU/L (SI: 43.6 IU/L) (reference, <2.0 IU/L [SI: <2.0 IU/L]); accordingly, thiamazole (MMI; 10 mg/day) was initiated at a prior facility. Combination therapy with MMI and levothyroxine was initially initiated due to low FT4 level, and both MMI and levothyroxine gradually tapered thereafter. Under medication of MMI (5 mg/day) and levothyroxine (12.5 μg/day), her thyroid function was favorably controlled (TSH, 2.90 μIU/mL [SI: 2.90 μIU/mL]; FT3, 2.8 pg/mL [SI: 4.30 pmol/mL]; FT4, 1.11 ng/dL [SI: 29.15 pmol/mL]; and TRAb, 3.5 IU/L [SI: 3.5 IU/L]) in 2017. During the period from August to October 2023, the patient exhibited persistently elevated TSH levels (7.49-11 μIU/mL [SI: 7.49-11 μIU/mL]) with normal FT3 (3.3-3.5 pg/mL [SI: 5.07-5.38 pmol/mL]) and FT4 values (1.11-1.57 ng/dL [SI: 14.29-20.21 pmol/mL]) under medication of MMI and levothyroxine at the same dose. After stopping both medications due to the favorable control of thyroid function in November 2023, laboratory evaluation at a prior hospital in January 2024 showed normal TSH level, high FT3 and FT4 levels (TSH, 3.46 μIU/mL [SI: 3.46 μIU/mL]; FT3, 5.0 pg/mL [SI: 27.35 pmol/mL]; FT4, 2.12 ng/dL [SI: 29.15 pmol/mL]), and low TRAb (0.80 IU/L [SI: 0.80 IU/L]) levels, suggesting inappropriate TSH secretion. Subsequently, a pituitary tumor was identified by magnetic resonance imaging (MRI) and she was referred to our department for further evaluation in February 2024. She had no family history. Her consciousness was alert. She had no neck goiter or leg edema. She had mild exophthalmos. She denied any headaches or visual field defects. Her physical findings were as follows: blood pressure, 127/75 mmHg; pulse, 105 beats/minute; body temperature, 36.8 °C; height, 163.7 cm; body weight, 53.8 kg; and body mass index, 20.1 kg/m^2^.

## Diagnostic Assessment

**Figure 1.**
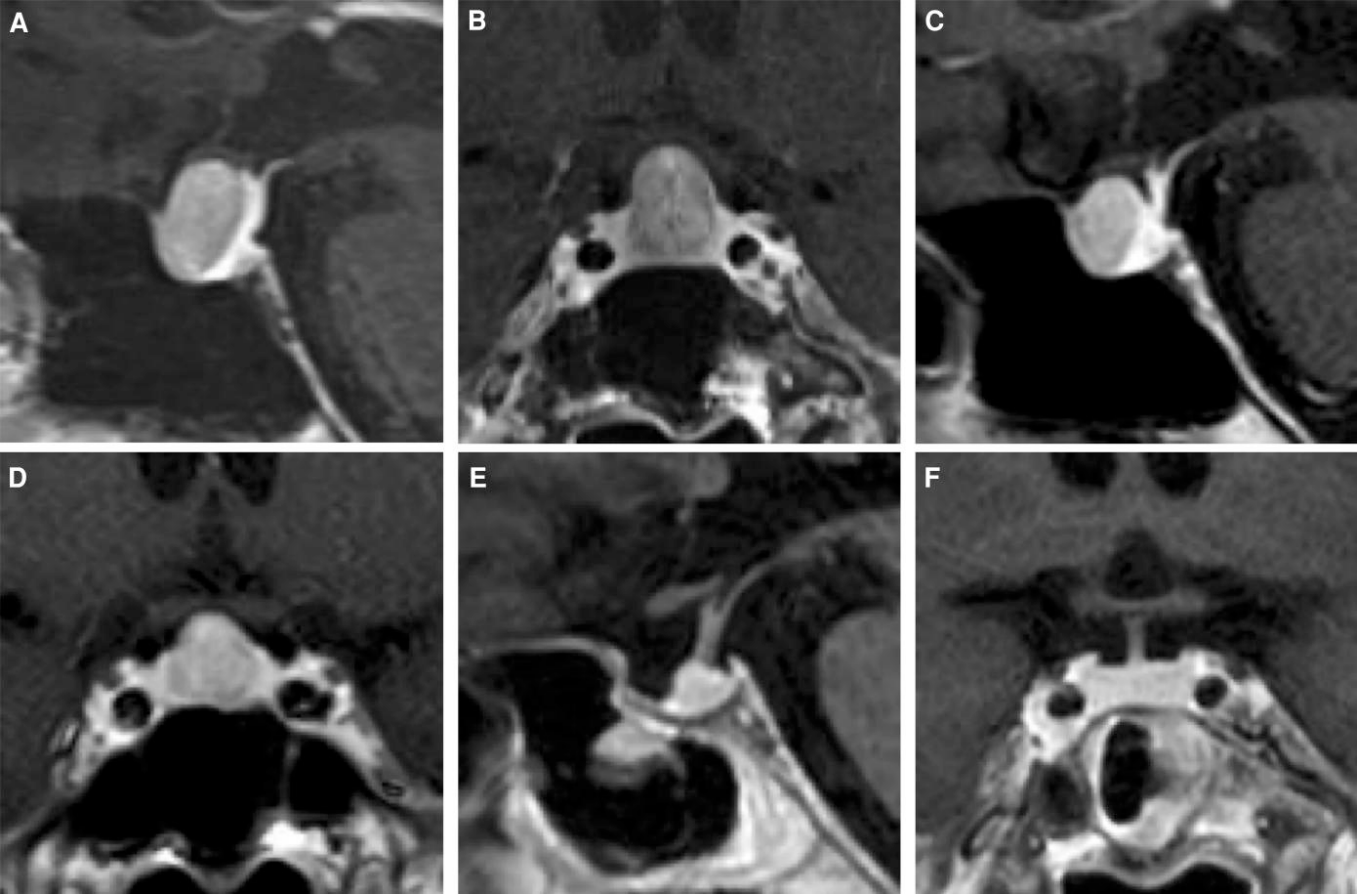
Magnetic resonance imaging (MRI) of the pituitary tumor across different stages of treatment. (A, B) Initial MRI with gadolinium enhancement. (A) Sagittal T1-weighted image showing the tumor. (B) Coronal T1-weighted image revealing tumor size (diameter was 10 × 12 × 13 mm) and morphology before treatment. (C, D) MRI after somatostatin analog injection with gadolinium enhancement. (C) Sagittal T1-weighted image showing the tumor shrinkage responded to treatment. (D) Coronal T1-weighted image demonstrating tumor size reduction. (E, F) Postoperative MRI with gadolinium enhancement following endoscopic transsphenoidal surgery. (E) Sagittal T1-weighted image showing the resection cavity. (F) Coronal T1-weighted image illustrating the residual normal pituitary tissue.

We reassessed the thyroid function in February 2024 and concluded inappropriate TSH secretion based on the results (TSH, 2.82 μIU/mL [SI: 2.82 μIU/mL]; FT3, 4.68 pg/mL [SI: 7.19 pmol/mL]; and FT4, 2.06 ng/dL [SI: 26.57 pmol/mL]). TSHoma is a known cause of inappropriate TSH secretion; delayed diagnosis could have led to tumor progression. Thus, this patient underwent brain MRI at our hospital, which revealed a gadolinium-enhanced tumor localized in the sella, anterior to the normal gland, with a maximum diameter of 13 mm (Fig. 1A and 1B). Blunted TSH responses to thyrotropin-releasing hormone stimulation have been seen in 90% of patients with TSHomas [1]. Regarding the thyrotropin-releasing hormone loading test using 0.2 mg of protirelin (Nipro ES Pharma, Osaka, Japan) intravenous injection, TSH levels showed a low response (from 1.61 to 2.80 μIU/mL [SI: from 1.61 to 2.80 μIU/mL]; cutoff, the peak TSH value is more than twice the baseline level) without any other pituitary dysfunctions. Considering the octreotide loading test using 50 mcg of octreotide (Novartis Pharma, Tokyo, Japan) subcutaneous injection, TSH levels showed a significant decrease (from 1.28 to 0.42 μIU/mL [SI: 1.28 to 0.42 μIU/mL]; cutoff, a reduction to less than 50% of the baseline value [2]). According to these findings, we diagnosed the patient with a TSHoma. Thyroid ultrasonography showed heterogeneous echogenicity but no nodule or increased blood flow.

## Treatment

**Figure 2.**
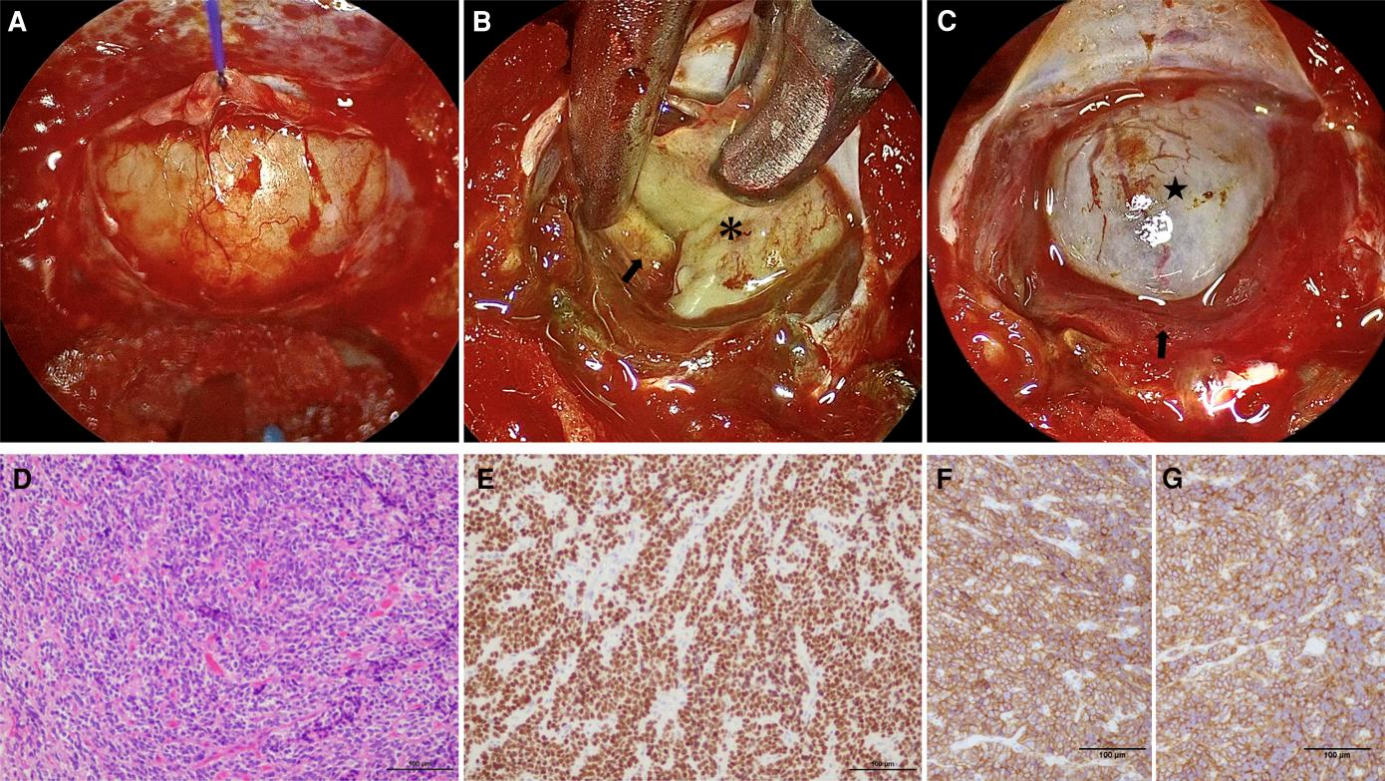
Surgical and pathological evaluation of the thyroid-stimulating hormone (TSH)-secreting pituitary adenoma. (A–C) Endoscopic surgical views. (A) Pre-resection view showing the tumor in situ. (B) Scene of tumor resection; the black arrow indicates the normal pituitary gland and the asterisk shows the tumor. (C) Postresection view showing the normal pituitary gland and arachnoid membrane after tumor removal; the black arrow indicates the normal pituitary gland and the star shows the arachnoid membrane. (D–F) Pathological evaluation of the resected tumor. (D) Hematoxylin and eosin (HE) staining demonstrating the cellular morphology of the tumor. (E) Immunohistochemical staining for TSH confirming the hormone production by the tumor. (F) Somatostatin receptor (SSTR)2 and (G) SSTR5 are expressed in the tumor cellular membrane.

To improve inappropriate TSH secretion (TSH, 2.82 μIU/mL [SI: 2.82 μIU/mL]; FT3, 4.68 pg/mL [SI: 7.19 pmol/mL]; and FT4, 2.06 ng/dL [SI: 26.57 pmol/mL]) during the perioperative period, the patient underwent a single subcutaneous injection of 90 mg of lanreotide acetate before the endoscopic endonasal surgery in February 2024. The syndrome of inappropriate secretion of TSH was immediately preoperatively normalized (TSH, 0.046 μIU/mL [SI: 0.046 μIU/mL]; FT3, 2.21 pg/mL [SI: 3.39 pmol/mL]; and FT4, 0.99 ng/dL [SI: 12.77 pmol/mL]; TRAb 1.2 IU/L [SI: 1.2 IU/L]), and the pituitary tumor showed preoperatively a slight shrinkage (Fig. 1C and 1D) in March 2024. In April 2024, we performed gross total resection of the tumor and removed the pseudocapsule of the tumor intervening between the normal gland and tumor by using an indocyanine green fluorescence 4K endoscope (Fig. 2A–2C) [3].

## Outcome and Follow-Up

**Figure 3.**
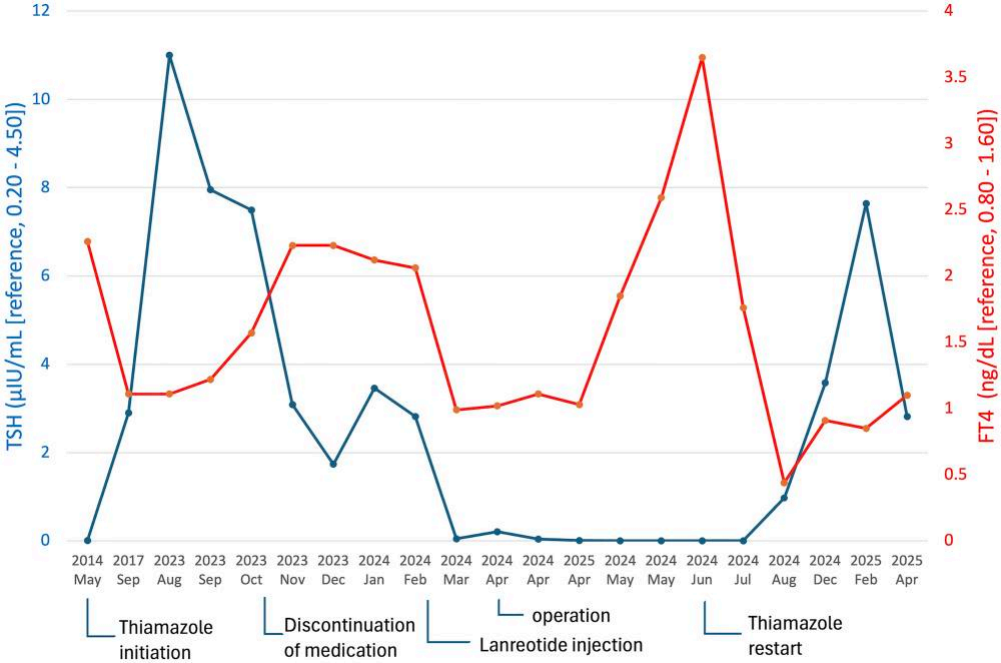
Changes in thyroid-stimulating hormone (TSH) and free thyroxine (FT4) levels during the treatment course. The graph illustrates the dynamic changes in TSH and FT4 levels over time in response to treatment. Initial medical therapy for Graves disease (GD) resulted in an increase in TSH levels and a decline in FT4 levels in 2014. Upon the cessation of oral medication in November 2023, FT4 levels rebounded with normal TSH levels, thus leading to a syndrome of inappropriate TSH secretion. After preoperative lanreotide injection in February 2024, the FT4 levels became normalized and inappropriate TSH secretion was resolved. However, the underlying GD state persisted in June 2024, 2 months later from endoscopic transsphenoidal gross total tumor resection, thus requiring ongoing management.

Histopathological examination of the tumor yielded a diagnosis of a pituitary adenoma on the basis of the dense population of round, short spindle-shaped atypical cells with anaerobic pigmented sporangia, as well as fibrotic stroma or vitellogeninic vessels and tumor cells positive for TSH and pituitary-specific transcription factor 1 on immunostaining. Additionally, the specimen was positive for both somatostatin receptors (SSTR)2 and SSTR5 (Fig. 2D–2G). Postoperatively, the patient demonstrated no other pituitary dysfunction. On postoperative day 5, TSH was 0.026 μIU/mL (SI: 0.046 μIU/mL), FT3 was 2.05 pg/mL (SI: 3.15 pmol/mL), and FT4 was 0.98 ng/dL (SI: 12.64 pmol/mL). However, she exhibited low TSH levels (<0.005 μIU/mL [SI: < 0.005 μIU/mL]), high FT4 levels (3.65 ng/dL [SI: 47.09 pmol/mL]), and high TRAb 3.9 IU/L [SI: 3.9 IU/L] in June 2024 (Fig. 3); therefore, MMI was restarted from 15 mg/day. Despite tapering MMI, she continues to take MMI (5 mg/day) and levothyroxine (75 mcg/day), involving the use of MMI as an antithyroid medication, with levothyroxine added due to decreased FT4 levels, which despite gross total resection of the tumor. Given that 2 months had passed since complete tumor resection, central hyperthyroidism due to TSHoma would be expected to have resolved, suggesting that the persistent hyperthyroid state was attributable to manifesting GD.

## Discussion

The coexistence of GD and a TSHoma is an exceptionally rare phenomenon. A few cases of plurihormonal pituitary adenoma [4] or mixed TSHoma [5] with GD have been reported. Primary hyperthyroidism, including GD, is characterized by high levels of serum thyroid hormones and low levels of TSH, which are suppressed by negative feedback. Regarding the pathophysiology, GD is considered an autoimmune disease in which antibodies stimulate TSH receptors on the thyroid gland, followed by increased thyroid hormones and decreased TSH levels through a negative feedback mechanism. Contrastingly, TSHoma is usually characterized by high levels of serum thyroid hormones but normal or mildly increased levels of serum TSH. MRI findings revealed the existence of a pituitary tumor, and it led to the diagnosis of a TSHoma coexisting with GD.

In cases of inappropriate TSH secretion, establishing a differential diagnosis between TSHoma and syndrome of resistance to thyroid hormone (RTH), which is a rare familial syndrome caused by thyroid hormone receptor beta (THRβ) disorder, is important. Most patients with RTH only require treatment of mild symptoms. However, patients with thyrotoxic symptoms, such as marked tachycardia, should be treated with beta blockers. Approximately 85% of patients with RTH have a THRβ genetic mutation, with the remaining patients having unclear etiology [6]. Recently, the association between RTH and autoimmune thyroid disease has been reported [7]. Checking the familial history and identification of a pituitary tumor on MRI is important. Additionally, patients with RTH show a low response to somatostatin analog compared with those with TSHoma; this difference can enhance the utility of differential diagnosis [2, 8]. In the present case, we made the diagnosis of a TSHoma because of the lack of relevant familial history, the response to somatostatin analog, and the detection of a pituitary tumor.

In this case, the diagnosis of TSHoma was delayed because the biochemical hallmarks of TSHoma were masked by the coexisting GD. As a result, the typical laboratory profile of a syndrome of inappropriate TSH secretion did not emerge until after the discontinuation of antithyroid therapy. In a patient with GD who presents with inappropriate TSH secretion despite the discontinuation of medication for GD, considering another undetected or overlapping condition with a different etiology, such as TSHoma, RTH, etc, is important [9, 10]. Previous reports have documented cases wherein GD was initially diagnosed, followed by the subsequent diagnosis of TSHoma [11-14]. In these reports, the longest interval between the diagnoses of both conditions was 4 years [14]. In contrast, the present case required 10 years to establish the coexistence of both diseases, making it the longest reported diagnostic interval compared with previous literature. The diagnosis of coexistence was challenging primarily because it took 10 years for inappropriate TSH secretion to be uncovered following the discontinuation of medication of GD.

The preoperative injection of somatostatin analog followed by surgical resection of TSHoma is effective in the tumor shrinkage and normalization of inappropriate TSH secretion [15]. Some studies have suggested that SSTR2 and SSTR5 expressions are clinically significant in the response to somatostatin analog [16, 17]. Additionally, a study showed that 3 of 16 patients (18.7%) with coexisting GD and TSHoma were on maintenance treatment of GD at the last follow-up [10]. This combined approach underscores the importance of a tailored treatment plan that addresses both pathologies [18]. The limitation of the present report is the absence of MRI evaluation during the treatment of GD throughout the clinical course.

In conclusion, the current case highlights the rarity of coexisting GD and TSHoma. The combination of biochemical analysis, antibody testing, and MRI of the pituitary region is essential for accurate and early diagnosis. Moreover, a multidisciplinary approach involving both surgical and medical treatments is necessary to address both pathologies. Given the rarity of such cases, further case studies and reviews would be benefit for clinical decision-making and optimization of patient outcomes.

## Learning Points

- The coexistence of a syndrome of inappropriate secretion of TSH due to a TSHoma and primary hyperthyroidism due to GD delayed the diagnosis of the former.
- Clinicians should be aware that the coexistence of GD and a TSHoma is a potential diagnostic consideration.
- A multidisciplinary approach involving both surgical and medical treatments is needed to address both pathologies.

## Data Availability

Original data generated and analyzed during this study are included in this published article.

## Abbreviations

FT3: free triiodothyronine
FT4: free thyroxine
GD: Graves disease
MMI: thiamazole
MRI: magnetic resonance imaging
RTH: resistance to thyroid hormone
SSTR: somatostatin receptor
TRAb: anti-thyrotropin receptor antibody
TSH: thyrotropin (thyroid-stimulating hormone)
TSHoma: thyroid-stimulating hormone–secreting pituitary adenoma
